# Guide Catheter-Induced Aortic Dissection Complicated by Pericardial Effusion with Pulsus Paradoxus: A Case Report of Successful Medical Management

**DOI:** 10.1155/2015/480242

**Published:** 2015-01-01

**Authors:** Magdalene Fiddler, Sriya A. Avadhani, Jonathan D. Marmur

**Affiliations:** ^1^Department of Internal Medicine, SUNY Downstate Health Science Center, 450 Clarkson Avenue, Brooklyn, NY 11203, USA; ^2^Department of Interventional Cardiology, SUNY Downstate Health Science Center, 450 Clarkson Avenue, Brooklyn, NY 11203, USA

## Abstract

Aortic dissection is a rare but potentially fatal complication of percutaneous coronary intervention (PCI). Management strategies of PCI induced dissection are not clearly identified in literature; such occurrences often mandate surgical repair of the aortic root with reimplantation of the coronary arteries. Another trend seen in case reports is the use of coronary-aortic stenting if such lesions permit. Several factors impact the management decision including the hemodynamic stability of the patient; mechanism of aortic injury; size, severity, and direction of propagation of the dissection; presence of an intimal flap; and preexisting atherosclerotic disease. We describe a case of a 65-year-old woman who underwent PCI for a chronic right coronary artery (RCA) occlusion, which was complicated by aortic dissection and pericardial effusion. Our case report suggests that nonsurgical management may also be appropriate for PCI induced dissections, and potentially even those associated with new pericardial effusion.

## 1. Introduction

Acute aortic dissection during cardiac catheterization has an estimated incidence of 0.02–0.04% [1, 2]; however, underreporting of this complication has been suspected. Iatrogenic aortic dissections occur more frequently in the setting of acute myocardial infarction (AMI) (0.19%) than for non-AMI interventions (0.01%) [1]. The incidence is generally higher during PCI (overall 0.03%) than during diagnostic procedures (<0.01%) in elective settings [3]. Aortic dissection may become immediately life threatening via a number of mechanisms, including (i) hemorrhage into the pericardium resulting in cardiac tamponade and hemodynamic collapse; (ii) occlusion of the contralateral coronary ostium (e.g., occlusion of the left main coronary artery during RCA PCI); (iii) occlusion of other aortic arch vessels, resulting in CVA; or (iv) propagation of the dissection into the descending aorta. The occurrence or potential for these complications may mandate surgical intervention, which carries a mortality risk of up to 25% [4].

Previous studies have identified risk factors for iatrogenic aortic dissections; however, these are limited to case reports due to the rarity of the event. Heavy calcifications in vessel walls that often require aggressive catheter manipulation, vigorous injection of contrast dye into the subintimal space, and usage of stiff wires and catheters that are wedged or in a noncoaxial position relative to the vessel wall have also been described [5, 6]. In addition, the use of Amplatz catheters has been frequently associated with ostial coronary artery dissection [1]. It also appears that RCA lesions are more frequently involved in iatrogenic aortic dissections [5]. This could be related to the fact that left main coronary arterial disease is very rarely an indication for PCI or due to a possible protective mechanism of the bigger ostium of left main vessel [5].

Iatrogenic dissections may involve different pathophysiologic mechanisms compared to spontaneous dissections that are secondary to atherosclerosis or connective tissue disorders. Aortic dissections that occur spontaneously have been associated with severe aortic pathology including degenerative changes of the medial layer and loss of elastic tissue [7]. These pathologic changes are not likely seen in PCI induced dissections; and therefore surgical management may be unnecessary to stabilize the patient's condition. Of the reported cases, iatrogenic aortic dissections are increasingly being managed by percutaneous stent placement or careful hemodynamic observation and medical therapy without surgery [5]. As described in our case, PCI induced aortic dissection even in the setting of hemodynamic compromise may be managed without surgical intervention.

## 2. Case Presentation

**Figure 1:**
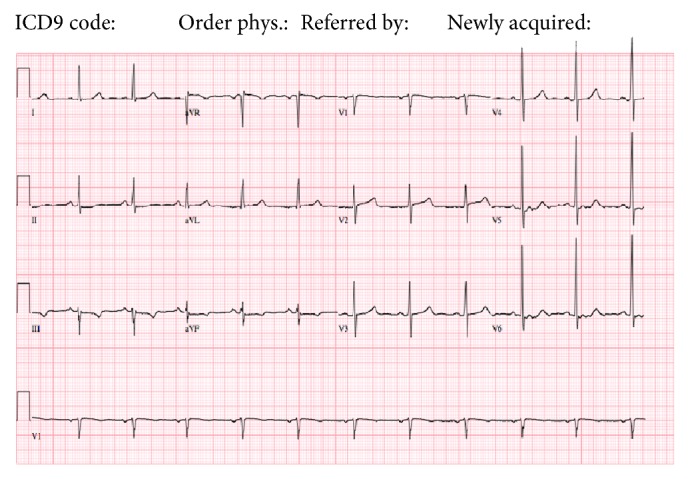
Admission EKG showing normal sinus rhythm (NSR) with T wave flattening and inversion in the inferior leads.

A 65-year-old African American woman with a history of hypertension and hyperlipidemia, who presented with a 2-month history of worsening pressure-like chest pain, was admitted for a non-ST-segment elevation myocardial infarction (NSTEMI). Serum troponin I peaked at 0.24 microgram/liter (*µ*g/L); electrocardiogram (ECG) showed nonspecific ST-T wave changes (Figure 1). Admission chest X-ray was normal.

**Figure 2:**
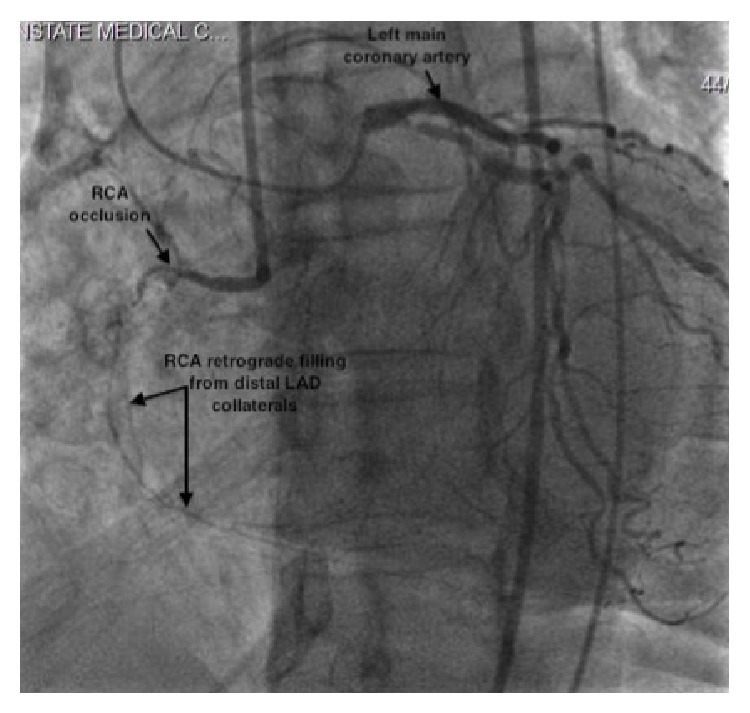
Diagnostic catheterization via simultaneous injection of the right and left coronary systems showing moderate collaterals from the distal LAD supplying the RCA and chronic total occlusion of the proximal RCA.

The patient was referred for cardiac catheterization and possible coronary intervention. Diagnostic cardiac catheterization revealed mild diffuse atherosclerotic disease of the left main and circumflex coronary arteries and chronic total occlusion of the proximal third of RCA with TIMI grade 0 flow [8]. The distal RCA filled via collateral arteries from the left anterior descending artery (LAD) and circumflex branches. Moderate collateral arteries from the septal LAD branches and distal circumflex supplied the distal right coronary artery (RCA) (see Figure 2).

The following day, in the setting of chest pain refractory to medical management the patient was referred for PCI of the RCA. Given the patient's presentation of worsening chest pain and troponin elevation, with minimal disease in the left coronary system, the proximal RCA occlusion was felt to be the lesion most likely responsible for the NSTEMI. She was sedated with midazolam 1 mg and fentanyl 25 mcg prior to the procedure. A 6FJR4 Brite Tip guiding catheter (Cordis, Bridgewater, NJ, USA) was used to cannulate the femoral artery, and a Whisper 0.014 × 190 cm wire (Abbott, Abbott Park, Illinois, USA) was used to cross the lesion in the RCA. A borderline successful balloon angioplasty was performed on the 100% lesion in the mid-RCA using a Maverick 2.0 × 9 mm balloon (Boston Scientific, Boston, MA, USA) with 5 inflations to a maximum inflation pressure of 6 atmospheres.

**Figure 3:**
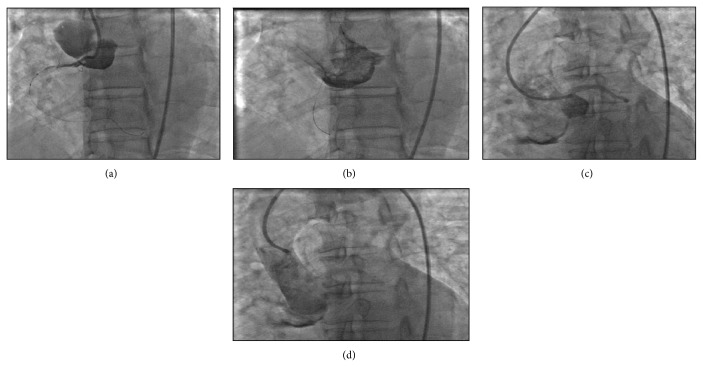
(a) Acute aortic dissection at the time of dye injection with the guide catheter and wire in place. (b) Visualization of the extent of dissection after injection of the dye. (c) Dye injection into left main coronary, no propagation to left coronary artery. (d) Aortogram showing dye staining of the aortic root dissection.

During the procedure the right guide catheter was changed to an Amplatz left-1 (Cordis, Miami, FL, USA) in order to provide more support for the introduction of devices into the coronary artery. This led to the dissection of the proximal RCA with retrograde extension into the aortic root and ascending aorta seen in Figure 3.

**Figure 4:**
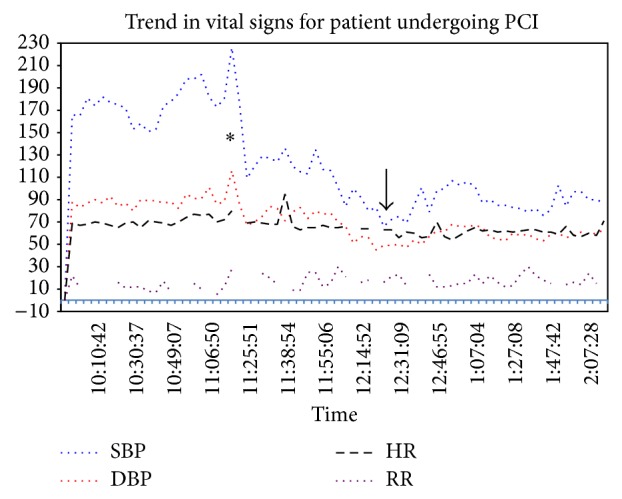
Trend in vital signs during acute aortic dissection. The time of dissection indicated by the asterisk, which is followed by the drop in systolic BP to around 60 (arrow) and the subsequent response to fluid resuscitation.

**Figure 5:**
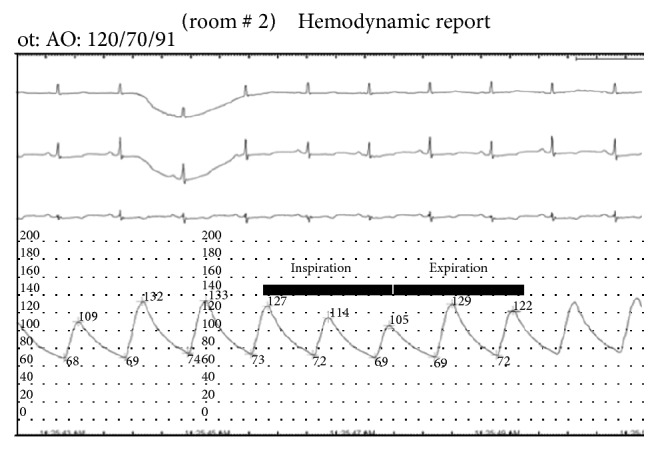
Blood pressure variation following aortic dissection with the inspiratory and expiratory phases denoted by the solid line. The inspiratory phase corresponds to a systolic blood pressure drop of about 22 mmHg.

Immediately following the dissection, a left coronary guiding catheter was used to define the status of the left main coronary ostium. Injection of dye into the aortic root with this catheter demonstrated patency of the left coronary system. Protamine sulfate was then administered for reversal of anticoagulation. There were brief episodes of hypotension with systolic blood pressure (SBP) dropping to 60–65 mmHg (Figure 4). During this time the patient was noted to have approximately 22 mmHg decrease in blood pressure during inspiration, consistent with pulsus paradoxus (Figure 5). She was immediately given a 1-liter bolus of normal saline resulting in an increased arterial blood pressure (Figure 4).

**Figure 6:**
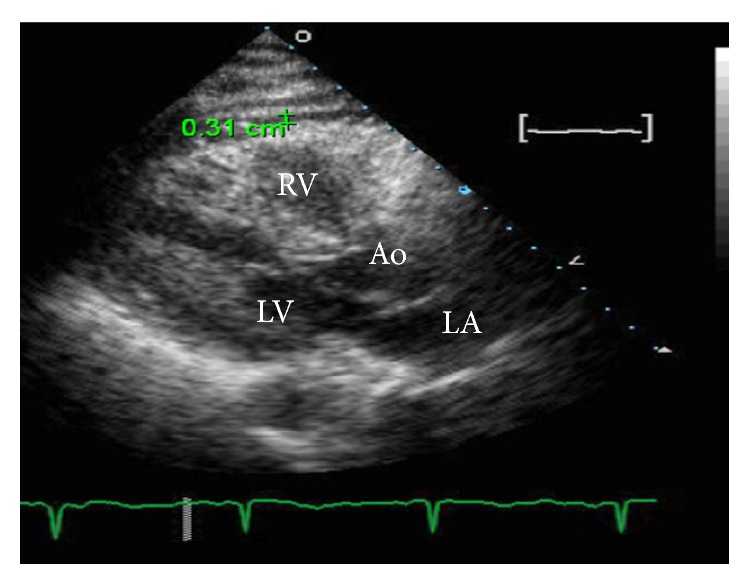
Echocardiographic evidence of pericardial effusion (measuring 0.31 cm) after aortic dissection on parasternal long axis view. Ao = aorta, RV = right ventricle, LV = left ventricle, and LA = left atrium.

On emergent echocardiography a small pericardial effusion was identified anterior to the right ventricle on the parasternal view measuring 0.31 cm (Figure 6). There were no echocardiographic signs of right atrial or right ventricular compression and no exaggerated respiratory variation in mitral flow velocities.

Cardiothoracic surgical consultation was immediately obtained to evaluate and prepare for an open repair of the aortic root and ascending aorta with reimplantation of the coronary arteries. During the time taken for surgical preparation, it was noted that the patient's vital signs stabilized to SBP 90–100 mmHg, pulse 55–70 beats per min (bpm), and respiratory rate (RR) 16–22/min with subjective improvement in chest pain. Due to the apparent improvement in hemodynamic status (systolic blood pressure following fluid challenge; lack of evidence of signs of tamponade on echocardiogram) of the patient, as well as no apparent angiographic propagation of the dissection, the decision was made to pursue medical management. The patient was then transferred to the recovery room for postintervention monitoring before being transferred to the coronary care unit (CCU).

**Figure 7:**
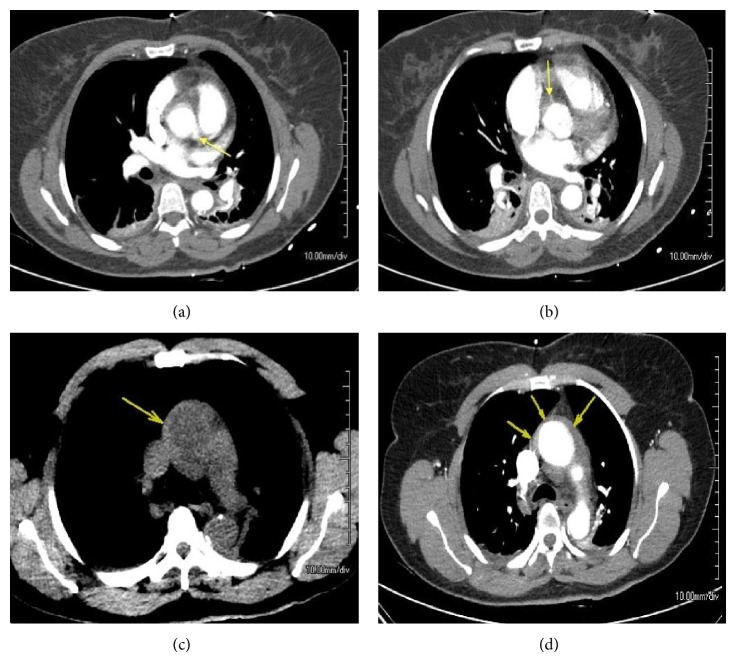
(a)-(b) CT angiogram at the level of the LCA and RCA, respectively (yellow arrows). (c)-(d) Arrows indicate the area of increased intensity suspicious for hemorrhage before (c) and after (d) contrast injection.

On arrival to the CCU, her vital signs remained stable, with SBP 120–130 mmHg, pulse 65 bpm with persistent nonprogressive chest pain. All anticoagulant, statin, and antiplatelet therapies were discontinued for the next 48 hours. The patient was started on nitroglycerine and esmolol drips (target SBP 100–110 mmHg and pulse 50–60 bpm) and then weaned off within the target range. She was then started on low dose beta-blocker, amlodipine, and isosorbide mononitrate therapy for maintenance. Computed tomography (CT) angiography scan was performed 12 hours after the PCI and showed no intimal dissection flap in the aorta. Along the ascending aorta up to the arch there was an increased surrounding density measuring roughly 30–40 HU, which was consistent with hemorrhage (Figures 7(c) and 7(d)). The descending thoracic aorta demonstrated luminal irregularity secondary to calcified mural plaques. The patient had an acute drop in hemoglobin from 12.5 to 10.2 grams/dL after procedure. Hence, CT of abdomen and pelvis was performed which ruled out retroperitoneal hemorrhage or other sources of intra-abdominal bleeding.

The patient had serum troponin I and ECG monitoring every 6 hours; troponin I peaked at 0.36 *µ*g/L with steady decline to <0.02 *µ*g/L over the next 24 hours. The patient had repeat echocardiography after procedure day 1, which was unchanged from her prior study. Chest radiography revealed bibasilar atelectasis without pleural effusion, and there was no evidence of mediastinal widening or any other radiographic evidence of aortic dissection.

The patient's antiplatelet medications were restarted 48 hours after procedure; antihypertensive medications were titrated to SBP 110–120 mmHg and pulse between 50 and 70 bpm. She was discharged home after procedure day 6, chest and epigastric pain free with follow-up within 1 week with the cardiology team. Upon follow-up, the patient continued to be chest pain-free; her EKG normalized and her vital signs were stable on medication to SBP around 120 and pulse 75 bpm. The patient was able to attend to all activities of daily living without difficulty.

## 3. Discussion

Aortic dissection is a rare but feared complication of PCI that is potentially fatal. Clear guidelines for the management of this complication are lacking, likely due to variations in severity, patient factors (e.g., medical history, age, and vascular anatomy), institution variables (e.g., procedural volume, operator experience, and high versus low-risk procedure), and experience in recognizing early signs of dissection.

Aortic dissections are generally described in terms of aortic arch and ascending aorta involvement (type A) versus that of the aorta distal to the right brachiocephalic artery and descending aorta (type B) [9]. Dissections involving the aortic arch and ascending aorta are generally viewed as life threatening warranting immediate surgical intervention while the latter can be managed medically. Although not standardized, Dunning et al. [1] have proposed a classification system for coronary artery provoked aortic dissections based on the extent of aortic involvement: Class I, where the contrast staining is limited to the coronary cusp; Class II, where contrast extends within 40 mm up the aortic wall; and Class III, where contrast extends to >40 mm up the aortic wall. While Classes I and II dissections are typically medically managed; Class III dissections may necessitate immediate surgical intervention and are associated with higher mortality. In this case, the dye staining on fluoroscopic view extended beyond the coronary cusp consistent with a Dunning Class II or III dissection with hemodynamic compromise, based on visual estimate. An accurate measurement of the length of dissection propagation was not made.

In the case of iatrogenic aortic dissection, several factors come into play when deciding what management approach should be employed. Factors that impact management decision include hemodynamic stability of the patient; mechanism of aortic injury; size, severity, and propagation of the dissection; presence of an intimal flap; and preexisting atherosclerotic disease. The mechanism of aortic injury during PCI assuming the patient has age-related atherosclerotic disease or hypertension, in the absence of collagen vascular disease for example, Marfan's and Ehlers-Danlos disease, is very different from that of spontaneous aortic dissection [10]. The former describes an externally applied mechanical force with trauma to an otherwise potentially normal vessel. In contrast, the latter describes a qualitative defect at cellular level in the infrastructure of the vessel. Because propagation of the dissection may be retarded by a more robust and healthy intrinsic architecture, it is not unreasonable to assume that patients with PCI induced dissection may benefit less from surgical intervention than patients presenting with spontaneous aortic dissection due to intrinsic vascular disease.

In the patient described, trauma caused by repeated attempts of guide-wire intubation may have caused perforation of the intima into the subintimal space of the RCA. In fact, the greatest risk factor for PCI-related dissections is the presence of heavily calcified vessels that often require aggressive catheter manipulation to deliver coronary stents and balloons [5]. Vigorous attempts to inject contrast, passage of stiff wires into the subintimal space, and use of guiding catheters have also been described [6]. In our case, the injection of dye may have contributed to the propagation of the dissection with retrograde extension to the posterior aortic root and ascending aorta. In patients with arterial dissection it may be prudent to avoid repeated and forceful dye injections. In one case reported by Sakakura et al., the use of ultrasound guided stent placement without the use of contrast in the presence of coronary-aortic dissections has been advocated [11].

Case reports [10, 12] identified the direction of the dissection as a prognostic indicator. Retrograde dissections frequently decrease or disappear relatively quickly, whereas anterograde dissections often remain patent for a longer period of time. In contrast to retrograde dissections, which extend in opposite direction to blood flow in the true lumen, anterograde dissections extend in the same direction of blood flow in the true lumen. This difference is understandable because blood pressure is pulsatile in an anterograde dissection but not in a retrograde dissection [12]. In our case there was a brief episode of hypotension that may have prevented further propagation of the dissection that can occur with pulsatile flow; in addition, prompt reversal of anticoagulation may have played a role in thrombosis of the false lumen.

A principle of management of iatrogenic aortic dissections is early recognition of hemodynamic instability such as cardiac tamponade. In this case there were early clinical signs of tamponade manifested by transient episodes of hypotension and pulsus paradoxus (Figures 3 and 4). There was echocardiographic evidence of pericardial effusion but no evidence of early hemodynamic compromise (no right atrial or right ventricular collapse). Another observation supporting the notion that this patient's pericardial pathology was not progressing to tamponade was her response to a 1-liter fluid bolus that led to elevation of her systolic blood pressure to above 100 mmHg. Our case report along with other published cases suggests that medical management of iatrogenic aortic dissections can result in successful outcomes, even in the presence of early signs of pericardial tamponade.

## 4. Conclusion

Management of complications associated with PCI requires rapid recognition, reversal and arrest of the underlying pathogenic mechanisms when possible (e.g., protamine to reverse anticoagulation, stent to prevent propagation), and selective surgical consultation in order to prevent morbidity and mortality. In the presence of low-risk dissections with limited damage to the aortic media, medical management with serial hemodynamic monitoring, imaging, and follow-up is appropriate. Our case report suggests that nonsurgical management may also be appropriate for PCI induced dissections and potentially even those associated with new pericardial effusion.

## Supplementary Material

Fluoroscopic video highlighting the Amplatz left-1 guide catheter induced proximal RCA dissection with extension into the aortic root and ascending aorta.
